# Supplementary material for: The Nursehound Scyliorhinus stellaris Mitochondrial Genome—Phylogeny, Relationships among Scyliorhinidae and Variability in Waters of the Balearic Islands
Source: Int J Mol Sci. 2022 Sep 8;23(18):10355. doi: 10.3390/ijms231810355 (PMC9499419; doi:10.3390/ijms231810355)
Supplement: Supplementary file 1 [file ijms-23-10355-s001.zip › ijms-1893902-supplementary.pdf]

Supplementary data

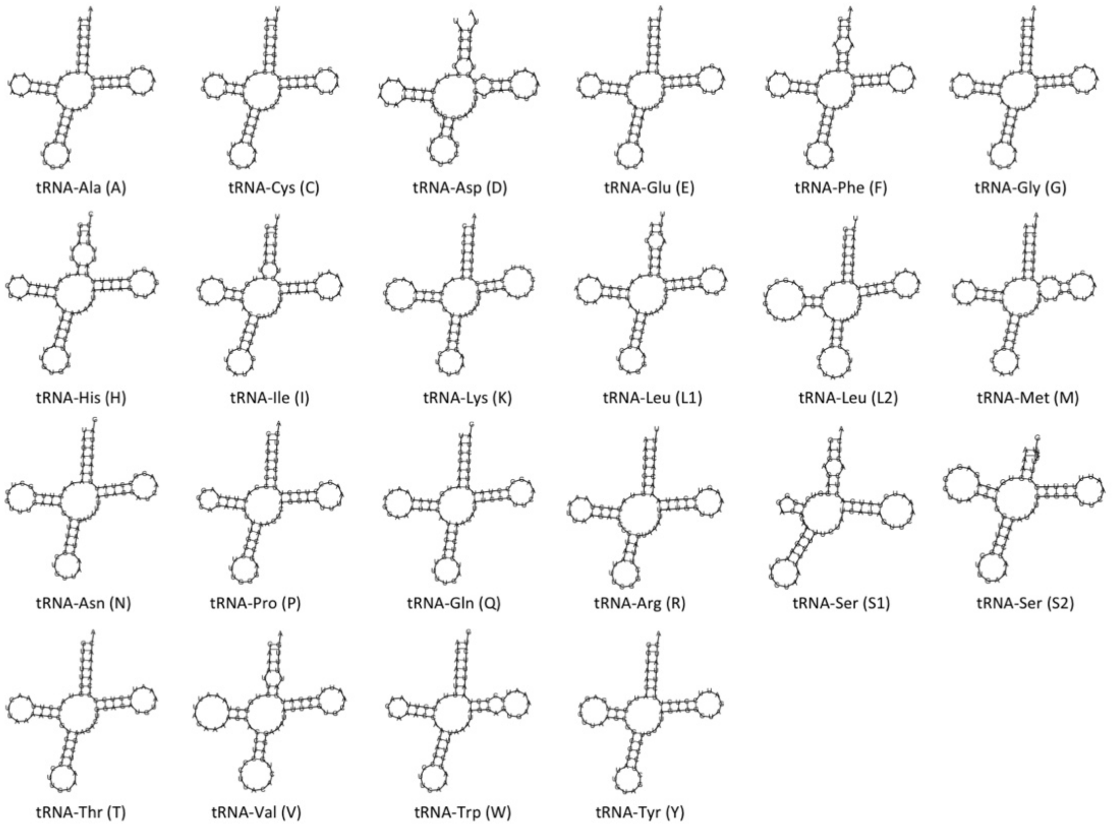

Figure S1 Putative secondary structure of the predicted 22 tRNAs based on the *S. stellaris* mitogenome sequence

	S. canicula				*S. stellaris vs. S. canicula* changes									
		**Codon**				**nucleotide position**								
	**Gene lenght**	**Start**	**Stop**	**AA**	**1st**	**2nd**	**3rd**	**overall**	**ts**	**tv**	**synon**	**nonsynon**	**n. AA change**	**% AA change**
COI	1554	GTG	TAA	517	10	3	93	106	84	22	98	8	6	1.16
COII	699	ATG	AGA	232	8	4	41	53	36	17	42	11	11	4.74
COIII	786	ATG	TAA	261	2	1	26	29	22	7	27	2	2	0.77
cytB	1144	ATG	T	381	18	5	68	91	75	16	74	17	17	4.46
ATP6	684	ATG	TAA	227	7	0	28	35	28	7	31	4	4	1.76
ATP8	168	ATG	TAA	55	2	1	7	10	9	1	6	4	4	7.27
ND1	975	ATG	TAA	324	16	4	68	88	72	16	79	9	8	2.47
ND2	1047	ATG	TAA	348	12	1	59	72	63	9	67	5	5	1.44
ND3	351	ATG	TAA	116	2	0	13	15	10	5	12	3	3	2.59
ND4	1381	ATG	T	460	18	8	73	99	82	17	80	19	17	3.70
ND4L	297	ATG	TAA	98	3	1	14	18	17	1	17	1	1	1.02
ND5	1830	ATG	TAA	609	22	6	99	127	109	18	108	18	16	2.63
ND6	522	ATG	TAG	173	5	5	40	50	45	5	39	9	6	3.47

	S. torazame				*S. stellaris vs. S. torazame* changes									
		**Codon**				**nucleotide position**								
	**Gene lenght**	**Start**	**Stop**	**AA**	**1st**	**2nd**	**3rd**	**overall**	**ts**	**tv**	**synon**	**nonsynon**	**n. AA change**	**% AA change**
COI	1554	GTG	TAA	517	26	1	162	189	143	46	180	9	7	1.35
COII	699	ATG	AGA	232	10	4	54	68	44	24	55	12	12	5.17
COIII	786	ATG	TAA	261	7	3	60	70	48	22	63	7	7	2.68
cytB	1146	ATG	TAG	381	40	10	123	173	125	48	130	43	35	9.19
ATP6	684	ATG	TAA	227	23	3	58	84	62	22	69	15	13	5.73
ATP8	168	ATG	TAA	55	8	2	14	24	20	4	16	8	6	10.91
ND1	975	ATG	TAA	324	18	2	106	126	87	39	111	15	13	4.01
ND2	1047	ATG	TAA	348	40	7	110	157	109	48	123	34	26	7.47
ND3	351	ATG	TAA	116	8	5	33	46	36	10	33	13	12	10.34
ND4	1381	ATG	T	460	38	16	136	190	141	49	142	48	41	8.91
ND4L	297	ATG	TAA	98	7	1	34	42	32	10	39	3	3	3.06
ND5	1830	ATG	TAA	609	45	14	190	249	188	61	196	52	41	6.73
ND6	522	ATG	TAA	173	12	12	67	91	63	28	66	24	18	10.40

	P. pantherinum				*S. stellaris vs.P. pantherinum* changes									
		**Codon**				**nucleotide position**								
	**Gene lenght**	**Start**	**Stop**	**AA**	**1st**	**2nd**	**3rd**	**overall**	**ts**	**tv**	**synon**	**nonsynon**	**n. AA change**	**% AA change**
COI	1554	GTG	TAA	517	17	1	154	172	137	35	165	7	6	1.16
COII	699	ATG	AGA	232	10	4	53	67	48	19	55	12	12	5.17
COIII	786	ATG	TAA	261	4	2	54	60	49	11	57	3	3	1.15
cytB	1144	ATG	T	381	30	4	103	137	107	30	110	27	23	6.04
ATP6	684	ATG	TAA	227	19	2	49	70	56	14	60	10	8	3.52
ATP8	168	ATG	TAA	55	4	2	11	17	15	2	10	7	7	12.73
ND1	975	ATG	TAA	324	16	2	93	111	88	23	101	10	10	3.09
ND2	1047	ATG	TAA	348	28	9	92	129	108	21	105	24	21	6.03
ND3	351	ATG	TAA	116	4	1	25	30	28	2	26	4	4	3.45
ND4	1381	ATG	T	460	40	9	115	164	133	31	126	38	34	7.39
ND4L	297	ATG	TAA	98	3	4	28	35	28	7	29	6	5	5.10
ND5	1830	ATG	TAA	609	39	18	165	222	193	29	180	41	39	6.40
ND6	519	ATG	TAA	172	11	11	42	64	48	16	42	22	19	11.05

	C. umbratile				*S. stellaris vs.C. umbratile* changes									
		**Codon**				**nucleotide position**								
	**Gene lenght**	**Start**	**Stop**	**AA**	**1st**	**2nd**	**3rd**	**overall**	**ts**	**tv**	**synon**	**nonsynon**	**n. AA change**	**% AA change**
COI	1554	GTG	TAA	517	23	1	147	171	135	36	164	7	6	1.16
COII	699	ATG	AGA	232	14	4	55	73	48	25	59	14	13	5.60
COIII	786	ATG	TAA	261	8	1	54	63	44	19	59	4	5	1.92
cytB	1146	ATG	TAG	381	29	8	129	166	124	42	136	30	27	7.09
ATP6	684	ATG	TAA	227	17	2	56	75	54	21	64	11	9	3.96
ATP8	168	ATG	TAA	55	8	2	14	24	19	5	16	8	5	9.09
ND1	975	ATG	TAA	324	21	2	95	118	88	30	105	13	11	3.40
ND2	1047	ATG	TAA	348	38	3	94	135	93	42	112	23	19	5.46
ND3	351	ATG	TAA	116	9	5	26	40	35	5	29	11	10	8.62
ND4	1381	ATG	T	460	48	12	128	188	146	42	139	49	42	9.13
ND4L	297	ATG	TAA	98	6	1	32	39	30	9	34	5	4	4.08
ND5	1830	ATG	TAA	609	40	14	185	239	183	56	192	46	37	6.08
ND6	522	ATG	TAA	173	16	11	58	85	63	22	56	27	23	13.29

	C. fasciatum				*S. stellaris vs.C. fasciatum* changes									
		**Codon**				**nucleotide position**								
	**Gene lenght**	**Start**	**Stop**	**AA**	**1st**	**2nd**	**3rd**	**overall**	**ts**	**tv**	**synon**	**nonsynon**	**n. AA change**	**% AA change**
COI	1554	GTG	TAA	517	18	1	151	170	136	34	164	6	11	2.13
COII	699	ATG	AGA	232	14	4	60	78	55	23	65	13	13	5.60
COIII	786	ATG	TAA	261	8	2	59	69	52	17	66	3	3	1.15
cytB	1147	ATG	TAG	381	29	6	124	159	123	36	133	26	24	6.30
ATP6	684	ATG	TAA	227	21	1	61	83	60	23	73	10	8	3.52
ATP8	168	ATG	TAA	55	8	2	14	24	22	2	16	8	5	9.09
ND1	975	ATG	TAA	324	21	1	91	113	86	27	103	10	10	3.09
ND2	1047	ATG	TAA	348	39	10	104	153	110	43	119	34	30	8.62
ND3	351	ATG	TAA	116	9	5	25	39	34	5	28	11	11	9.48
ND4	1381	ATG	T	460	43	13	152	208	169	39	166	42	42	9.13
ND4L	297	ATG	TAA	98	5	1	35	41	32	9	37	4	4	4.08
ND5	1830	ATG	TAA	609	50	11	179	240	187	53	191	48	39	6.40
ND6	522	ATG	TAA	173	11	8	53	72	54	18	51	20	16	9.25

Table S1 Nucleotide substitutions and Amino acid changes in Scyliorhininae species versus *S. stellaris*

	Carcharhinus_acronotus	Carcharhinus_amblyrhynchoides	Carcharhinus_amboinensis	Carcharhinus_falciformis	Carcharhinus_obscurus	Carcharhinus_melanopterus	Carcharhinus_sorrah	Eusphyra_blochii	Galeocerdo_cuvier	Galeus_melastomus	Glyphis_gangeticus	Halaelurus_buergeri	Hemigaleus_microstoma	Lamiopsis_tephrodes	Loxodon_macrorhinus	Mustelus_mustelus	Prionace_glauca	Proscyllium_habereri	Scoliodon_laticaudus	Sphyrna_lewini	Triaenodon_obesus	Poroderma_pantherinum	Scyliorhinus_canicula	Scyliorhinus_torazame	Scyliorhinus_stellaris	Cephaloscyllium_fasciatum	Cephaloscyllium_umbratile
Carcharhinus_acronotus																											
Carcharhinus_amblyrhynchoides	0.0864																										
Carcharhinus_amboinensis	0.0831	0.0888																									
Carcharhinus_falciformis	0.0790	0.0870	0.0850																								
Carcharhinus_obscurus	0.0700	0.0795	0.0758	0.0696																							
Carcharhinus_melanopterus	0.0895	0.0804	0.0930	0.0901	0.0857																						
Carcharhinus_sorrah	0.0827	0.0879	0.0868	0.0812	0.0689	0.0961																					
Eusphyra_blochii	0.1436	0.1448	0.1383	0.1421	0.1399	0.1462	0.1412																				
Galeocerdo_cuvier	0.1513	0.1505	0.1492	0.1502	0.1442	0.1530	0.1570	0.1606																			
Galeus_melastomus	0.2032	0.1979	0.2021	0.2011	0.2030	0.2033	0.2023	0.2098	0.2027																		
Glyphis_gangeticus	0.1208	0.1170	0.1186	0.1177	0.1097	0.1225	0.1145	0.1516	0.1635	0.2131																	
Halaelurus_buergeri	0.2201	0.2191	0.2149	0.2230	0.2230	0.2162	0.2185	0.2298	0.2337	0.2055	0.2343																
Hemigaleus_microstoma	0.1731	0.1680	0.1671	0.1723	0.1721	0.1717	0.1709	0.1747	0.1889	0.2248	0.1754	0.2352															
Lamiopsis_tephrodes	0.1172	0.1222	0.1154	0.1181	0.1143	0.1273	0.1170	0.1478	0.1647	0.2144	0.0911	0.2332	0.1774														
Loxodon_macrorhinus	0.1393	0.1436	0.1430	0.1430	0.1412	0.1443	0.1410	0.1522	0.1705	0.2033	0.1535	0.2267	0.1743	0.1472													
Mustelus_mustelus	0.1579	0.1556	0.1629	0.1542	0.1537	0.1586	0.1506	0.1616	0.1640	0.1841	0.1668	0.2186	0.1720	0.1659	0.1570												
Prionace_glauca	0.0939	0.1010	0.1006	0.0778	0.0868	0.1044	0.0988	0.1507	0.1596	0.1985	0.1325	0.2223	0.1746	0.1324	0.1565	0.1635											
Proscyllium_habereri	0.1813	0.1764	0.1816	0.1815	0.1754	0.1793	0.1789	0.1894	0.1909	0.1787	0.1914	0.2136	0.2027	0.1904	0.1807	0.1655	0.1851										
Scoliodon_laticaudus	0.1764	0.1753	0.1755	0.1819	0.1770	0.1796	0.1746	0.1822	0.1834	0.2082	0.1849	0.2295	0.2052	0.1810	0.1596	0.1858	0.1827	0.1920									
Sphyrna_lewini	0.1393	0.1407	0.1426	0.1380	0.1396	0.1413	0.1416	0.1190	0.1705	0.2139	0.1455	0.2234	0.1733	0.1479	0.1513	0.1703	0.1491	0.1899	0.1862								
Triaenodon_obesus	0.0832	0.0965	0.0883	0.0897	0.0795	0.1002	0.0897	0.1511	0.1630	0.2085	0.1253	0.2302	0.1737	0.1266	0.1500	0.1629	0.1072	0.1843	0.1826	0.1459							
Poroderma_pantherinum	0.2197	0.2228	0.2251	0.2247	0.2208	0.2186	0.2180	0.2208	0.2276	0.2147	0.2245	0.2378	0.2335	0.2287	0.2156	0.2146	0.2234	0.2047	0.2237	0.2192	0.2211						
Scyliorhinus_canicula	0.2205	0.2257	0.2286	0.2281	0.2201	0.2268	0.2212	0.2268	0.2319	0.2170	0.2318	0.2412	0.2364	0.2255	0.2216	0.2153	0.2226	0.2072	0.2314	0.2301	0.2265	0.1270					
Scyliorhinus_torazame	0.2372	0.2376	0.2414	0.2427	0.2397	0.2355	0.2374	0.2423	0.2468	0.2318	0.2452	0.2446	0.2540	0.2426	0.2355	0.2287	0.2347	0.2126	0.2423	0.2486	0.2396	0.1571	0.1484				
Scyliorhinus_stellaris	0.2177	0.2269	0.2258	0.2216	0.2161	0.2258	0.2175	0.2221	0.2267	0.2134	0.2282	0.2404	0.2401	0.2251	0.2251	0.2116	0.2226	0.2054	0.2280	0.2319	0.2245	0.1262	0.0743	0.1503			
Cephaloscyllium_fasciatum	0.2282	0.2263	0.2266	0.2268	0.2247	0.2229	0.2246	0.2314	0.2389	0.2225	0.2312	0.2397	0.2433	0.2360	0.2320	0.2183	0.2253	0.2085	0.2378	0.2371	0.2290	0.1491	0.1432	0.1266	0.1439		
Cephaloscyllium_umbratile	0.2234	0.2191	0.2172	0.2253	0.2223	0.2195	0.2217	0.2234	0.2306	0.2179	0.2264	0.2399	0.2388	0.2240	0.2248	0.2225	0.2269	0.2052	0.2304	0.2342	0.2226	0.1500	0.1426	0.1348	0.1402	0.1119	
Isurus_oxyrinchusOutgroup	0.2602	0.2580	0.2632	0.2588	0.2608	0.2592	0.2542	0.2535	0.2706	0.2784	0.2526	0.2835	0.2636	0.2634	0.2589	0.2605	0.2653	0.2663	0.2770	0.2509	0.2552	0.2576	0.2672	0.2791	0.2676	0.2724	0.2736

Table S2 Pairwise genetic distance among Carchariniformes species

Forward	5'-3'	Reverse	5'-3'
ARN16S1	CCTCGCCTGTTTACCAAAAACATCGCCTC	ARN12Sec4	GCGGTGGCTGGCACGAGTTTTACC
Scy16sec1	CCTAGGGATAACAGCGCAAT	Scy12Ssec2	TCTTTCCACTTCATTCGCTACACCT
ScyND2sec1	CCCCAGGCCCCATTAATATAGCATC	ARN12S2	TGCACCTTCCAGTACACTTACCATGTTACGAC
ScyCOIsec1	TCCTGTCCTTGCAGCCGGAA	ARN16S2	TAATAGCGGCTGCACCATTAGGATGTCCTG
ScyCOIIsecF	CATAGTTGTACCCATAGAATCCCCT	ScyND2sec2	CACCTGAAGTCCAAGCGTT
ScyFor1	AAACTATCAGCTTATTTATTCGACCAT	ScyCOIsec2	ACCTAAAAGTGAACCCGGCTGACC
ScyFor3	CTTTGAAGCTGCCGCATGATACTGAC	ScyCOIIsec2	TGCCATTAAAATAAGATACGGGAG
ScyFor5	AATCAAACAGAACGCCTAAATGCAG	ScyRew2	GCGATTAATTGTATAAGAAGGTGACCA
ScyFor7	CTTAACCCTTCTTACACACGAGA	ScyRew4	TGAGCCTCATCAATAAATAGACACA
ScyFor11	GACCCTCAAGACAACCCACGAGT	ScyRew6	TACAAGGGTATTTTAACTAGGAAGGCAAT
ScyFor13	TTTCCAACCCATTTAATTCCTCAACACC	ScyRew8	AATGGATCAGGTAACATAAAGGGCAAC
ScyCytb1	TTCTACCTTGAGGCCAAATATCCTTC	ScyRew12	AATCCTTCTCCTTATTATGCGGCTT
ScyPro1	AATTCTGCCTAAACTGCCCTCT	Scycytb2	ATGGTTGTTCTACTGGTTGTCCTCCAA
ScyDloop3F	GGTACACCCAAATCTTTAGTGCAT	ScyDloop4R	ATGTATATATTAAGGTATGTGGGCTATGTCA
		ScyDloop2R	AAAATAATATATGTCGGCCCTCGTT

TableS3 Primers for *S. stellaris* mitogenome amplifications
